# Early-Life Experiences as Moderators of the Relationship Between Extreme Heat and Alcohol Consumption among Older Adults: Quantitative Study

**DOI:** 10.2196/76904

**Published:** 2026-06-10

**Authors:** Hee Yun Lee, Su Hyun Shin, Yeon Jin Choi, Eun Young Choi, Hyunjung Ji

**Affiliations:** 1 School of Social Work, University of Georgia, 279 Williams Street, Athens, GA, 30602, United States, 1 (706) 542-3364; 2 Department of Family and Consumer Studies, University of Utah, Salt Lake City, UT, United States; 3 College of Social Work, University of Kentucky, Lexington, KY, United States; 4 School of Gerontology, University of Southern California, Los Angeles, CA, United States; 5 Department of Political Science, The University of Alabama, Tuscaloosa, AL, United States

**Keywords:** alcohol use, adverse childhood experiences, ACEs, parental substance abuse, life course perspective framework, psychosocial vulnerability

## Abstract

**Background:**

Climate change has intensified the frequency and duration of extreme heat events worldwide, posing growing public health risks, particularly for older adults who are physiologically more susceptible to heat-related illnesses. Concurrently, alcohol consumption among older adults in the United States has risen significantly over the past 2 decades, increasing vulnerability to dehydration, cardiovascular strain, and cognitive impairment during heat exposure. Emerging evidence suggests that environmental stressors such as extreme heat may exacerbate maladaptive coping behaviors, including alcohol use; however, few studies have examined this association in aging populations. Moreover, little is known about how early-life experiences such as childhood adversity or positive parental relationships shape behavioral responses to environmental stressors later in life.

**Objective:**

This study examines the relationship between extreme heat and alcohol consumption among older Americans, emphasizing the moderating role of early-life experiences within a life course framework.

**Methods:**

Using data from individuals aged >50 years in the Health and Retirement Study (1996‐2018), we analyzed the association between extreme heat exposure (>95 °F) and alcohol consumption, while examining whether early-life experiences such as parental substance abuse, law enforcement encounters, and relationships with fathers moderate this relationship.

**Results:**

Extreme heat exposure was significantly associated with increased alcohol consumption (0.21% per additional extreme heat day, *P*<.001). A positive father-child relationship buffered this effect, while adverse early-life experiences, including law enforcement encounters (0.08%, *P*<.001) and parental substance abuse (0.05%, *P*<.001), exacerbated it.

**Conclusions:**

Given the link between extreme heat and alcohol use in older adults, further longitudinal research and targeted interventions are needed to mitigate associated health risks. Strengthening positive childhood experiences may offer long-term protective effects by fostering resilience and healthier coping mechanisms that persist into later life.

## Introduction

### Background

The increasing prevalence of alcohol consumption, particularly high-risk and excessive drinking, among older adults has emerged as a growing public health concern in the United States [1-4]. Between 2001 and 2013, there was a marked rise in alcohol use patterns among US adults aged ≥65 years, characterized by a 22% increase in past-year alcohol consumption, a 65% increase in high-risk drinking, and a staggering 107% increase in alcohol use disorder diagnoses [2]. More recent data reveal that over half of adults in this age group reported alcohol consumption within the past year, with approximately 11% engaging in binge drinking within the past month [5]. These trends are particularly alarming, given the heightened susceptibility of older adults to alcohol-related health complications, owing to age-related physiological changes, coexisting chronic conditions, and potential interactions with medications—factors that collectively elevate risks for unintentional injuries, toxic reactions, and exacerbation of preexisting health conditions [6,7]. As the aging population continues to grow, so too will the public health implications of alcohol consumption among older adults, underscoring the urgent need for risk mitigation and intervention strategies.

### Extreme Heat Exposure and Alcohol Consumption

While previous research has predominantly centered on individual-level determinants of alcohol use among older adults, such as demographic characteristics, socioeconomic status, and health conditions [3-8], a growing body of literature highlights the influence of environmental factors, particularly climate-related stressors such as extreme heat. The hypothesis that climate change–induced extreme heat may exacerbate substance use disorders is gaining empirical support [9]. Scoping reviews suggest that climate change contributes to the worsening of mental health conditions, including heightened levels of anxiety, depression, and psychological distress [10], which may in turn lead to increased substance use as a maladaptive coping mechanism. Notably, empirical evidence from Shenzhen, China, the first study to investigate the relationship between extreme heat and acute excessive drinking, identified a positive correlation between heat exposure and the likelihood of engaging in heavy drinking episodes [11]. Similarly, a nationwide survey in Australia reported that 11% of respondents consumed more alcohol on extremely hot days [11]. Despite these important contributions, prior studies have not specifically targeted older adults nor used nationally representative samples, leaving critical gaps in understanding how heat exposure may influence alcohol consumption patterns in aging populations.

Several physiological and psychological mechanisms may explain the link between high temperatures and increased alcohol use. On a physiological level, alcohol can alleviate thermal discomfort by promoting vasodilation, enhancing perspiration, and facilitating heat dissipation [12,13]. Cold carbonated alcoholic beverages, such as beer, may further provide immediate relief and perceived hydration [14]. Psychologically, alcohol’s mild anxiolytic and analgesic properties may offer temporary relief from heat-induced discomfort, irritability, or anxiety [15,16]. However, relying on alcohol as a coping strategy during heatwaves may pose serious health risks, exacerbating dehydration, impairing thermoregulation, and amplifying the risk of heat-related illnesses [11].

### Early-Life Experiences as Moderators

Importantly, individual susceptibility to environmental stressors such as extreme heat is not uniform. Psychosocial resources and early-life experiences play a pivotal role in shaping vulnerability and resilience. The existing literature underscores that climate-related health risks are not solely determined by exposure intensity but are also mediated by underlying vulnerability factors (ie, predisposition to adverse outcomes) and resilience capacities [17]. While studies have highlighted personal socioeconomic status, education, income, and social isolation as key determinants of resilience [18], the influence of early-life experiences, both positive childhood experiences (PCEs) and adverse childhood experiences (ACEs), remains underexplored in this context.

PCEs such as parental care, emotional support, and stable family relationships foster resilience and serve as protective factors for mental health in later life [19,20]. For instance, receiving unconditional love and experiencing nurturing parenting contribute to enhanced coping skills, reducing reliance on harmful behaviors such as substance abuse [21]. Conversely, ACEs such as exposure to parental substance misuse, neglect, abuse, or encounters with law enforcement are strongly associated with heightened psychosocial vulnerability and maladaptive coping strategies, including alcohol abuse, in adulthood [22-25]. There is extensive evidence linking ACEs to increased risk of mental health challenges such as depression, psychiatric disorders, and substance misuse later in life [26-29]. Early adverse experiences may thus compound the impact of environmental stressors, amplifying health risks through entrenched behavioral and psychological pathways.

### This Study

Grounded in a life course perspective, this study aims to investigate the relationship between extreme heat exposure and alcohol consumption among older adults in the United States, with a focus on how early-life experiences shape risk disparities. Leveraging over 2 decades of nationally representative data integrated with historical temperature records, we provide a comprehensive analysis of this association, addressing a critical gap in the literature. While previous research has predominantly emphasized adulthood socioeconomic and psychosocial factors, our study extends this framework by elucidating how both PCEs and ACEs moderate older adults’ behavioral responses to extreme heat. This inquiry is particularly timely given the projected rise in the frequency and severity of heatwaves associated with climate change [30].

The intersection of extreme heat and alcohol consumption is a public health issue of growing urgency, as alcohol use during heatwaves may exacerbate vulnerability to acute health crises, including dehydration, hyperthermia, and heat stroke [31,32]. Older adults, already facing age-related declines in thermoregulatory capacity, are at heightened risk. By identifying how early-life experiences shape susceptibility to maladaptive coping behaviors under environmental stress, our findings offer actionable insights for designing targeted interventions and health policies aimed at mitigating alcohol-related harms in the context of climate change. To our knowledge, this is the first study to use a nationally representative dataset to explore the intersection of extreme heat exposure, substance use, and early-life experiences among older adults in the United States.

## Methods

### Data and Sample

As individual-level data, this research used data collected from a nationally representative sample of the Health and Retirement Study (HRS), sampling over 20,000 older adults aged ≥50 years, along with their spouses, living in the United States. Since 1992, the HRS has conducted biennial surveys. This comprehensive survey covers diverse information, including demographics, physical health, health-related behaviors, family dynamics, disabilities, housing, wealth, work history, health care accessibility, and more [33]. This study used the 1996 to 2018 waves of the HRS.

The National Environmental Public Health Tracking Network, a Center for Disease Control and Prevention division, offers yearly county-level data concerning extreme weather incidents. This data source provides historical and projected metrics linked to temperature, heat index, heat-related ailments, and previous and predicted precipitation. To explore the relationship between heat events and the drinking behaviors of older adults, we merged the county-level data on extreme weather incidents with the individual-level data of the HRS using the participant’s county of residence and the years in which the survey was conducted.

The study included participants aged ≥50 years with geographical information about their county of residence across time. The detailed sample characteristics are presented in Table 1. The average age of the sample was 68 (SD 11) years. More than half of the sample were women (57%), non-Hispanic White (64%), and coupled (61%). Approximately two-thirds of the respondents completed a high school degree or lower (76%), were unemployed or retired (73%), and owned a primary residence (74%). A total of 65% of the older adults reported good or better health, and <1% reported activity of daily living and instrumental activity of daily living with difficulty. The mean number of household members and living children was 2 (SD 1) and 3 (SD 2), respectively.

**Table 1.** Sample characteristics.^a^

Variables	Participants
Outcome variable, mean (SD)	
Drinks per week	0.38 (2.97)
Explanatory variables, mean (SD)	
Extreme heat days ≥95	16.63 (30.43)
Extreme heat days ≥100	7.18 (19.15)
Extreme heat days ≥105	2.44 (9.03)
Moderators: early-life experiences	
Relational quality with father, mean (SD)	4.21 (1.18)
Police encounter (%)	5.28
Parental drink or drug use (%)	16.08
Covariates	
Age (years), mean (SD)	68.19 (11.12)
Gender (%)	
Men	42.70
Women	57.30
Race or ethnicity (%)	
Black	19.58
Hispanic	12.69
Other	3.78
White	63.95
Education (%)	
Less than high school	25.03
High school	50.66
Some college	5.27
Bachelor of Arts	12.03
Graduate	7.01
Marital status (%)	
Coupled	60.68
Separated, divorced, or widowed	34.97
Never married	4.36
Self-reported health (%)	
Poor	10.91
Fair	23.73
Good	31.77
Very good	24.84
Excellent	8.75
ADLs^b^, mean (SD)	0.49 (1.13)
IADLs^c^, mean (SD)	0.45 (1.11)
Health insurance (%)	
Yes	93.22
No	6.78
Employment status (%)	
Employed	27.29
Unemployed	14.74
Retired	57.97
Homeownership (%)	
Yes	73.76
No	26.24
Household income (US $), mean (SD)	62,372 (228,780)
Financial assets (US $), mean (SD)	159,621 (813,402)
People in a household, mean (SD)	2.27 (1.29)
Living children, mean (SD)	3.20 (2.15)
Extremely rainy days, mean (SD)	7.78 (4.87)

a Unweighted estimates. 1996 to 2018 waves of the Health and Retirement Study (N=35,261; observations [respondent wave], observations=148,696, N=148,696).

b ADL: activities of daily living.

c IADL: instruments of activities of daily living.

### Variables

The study’s dependent variable was older adults’ alcohol consumption behaviors measured using the natural log-transformed number of drinks consumed per week. To create the number of drinks taken per week, the study combined two questions asking how many days per week a respondent has had any alcohol to drink and how many drinks she had on the days of drinking in the previous 3 months. Responses to these questions were multiplied. The average number of alcoholic beverages consumed in a week was estimated as <1 (Table 1). The ratio of observations who had at least 1 drink per week was 5%. Among drinkers, the mean number of drinks consumed per week was estimated as 7.85 (SD 11.06).

The primary explanatory variable in the study was instances of extreme heat events taking place in the HRS respondents’ county. This factor was proxied by examining the annual frequency of extreme heat days, by counting the days when the highest daily temperatures reached 95 °F, 100 °F, and 105 °F between May and September. On average, there were 17 days per year with peak temperatures of 95 °F, 7 days per year with peak temperatures of 100 °F, and 2 days per year with peak temperatures of 105 °F (Table 1).

The study considered early-life experiences as a moderator of the relationship between extreme heat days and alcohol consumption. The early-life events included paternal relational quality, police encounters, and parental substance use problems before age 18 years, all of which were included in the Leave Behind section of the HRS. Since 2006, 50% of the main survey participants have randomly been assigned to complete the Leave Behind section, and the other half was required to do so in 2008, resulting in respondents’ participation in the section in 4-year intervals. The paternal relational quality before age 18 years was assessed using the question, “I had a good relationship with my father before age 18,” rated on a 5-point Likert scale [34]. The mean score was 4.21 (SD 1.18), with higher values indicating better relationship quality. As the measure asked about early relational quality, the measure was also averaged across waves to have a time-invariant individual factor. The score was further standardized to have a mean of 0 (SD 1). The other early-life events associated with later-life alcohol consumption were included: “Before you were 18 years old, were you ever in trouble with the police?” “Before you were 18 years old, did either of your parent drink or use drugs so often that it caused problems in the family?” [35]. The percentage of the observations that encountered law enforcement and had parents with drinking and drug use problems was 5.28% and 16.06%, respectively. The two variables were also coded as time-invariant factors, using the mode of the responses.

The study included a comprehensive set of time-variant individual and household characteristics as covariates. These include age centered on 50 years, its squared term, marital status, self-reported health, number of activities of daily living and instrumental activities of daily living with difficulty performing, health insurance ownership, employment status, home ownership, the natural logarithm of household income and financial assets, and number of household members and living children. The study further controlled for individual and wave fixed effects. The study finally controlled for the yearly number of days experiencing intense precipitation, quantified by the annual heavy rainfall exceeding the absolute threshold of 0.01 inches. The study found that, on average, there were 8 such extreme precipitation days per year during the study period.

### Empirical Models

To estimate the effect of extreme heat events on alcohol consumption behaviors, this study used individual fixed effects models. By controlling for individual fixed effects, the models were designed to capture within-individual changes in behaviors in response to changes in extreme heat events. The following is the specified model:


(1) $$AC_{iw}=\alpha_{0}+\alpha_{1}EH_{ct}+\alpha_{2}X_{iw}+i_{i}+w_{w}+\epsilon_{iw}$$

*AC_iw_* denotes a respondent *i*’s alcohol consumption behaviors measured using the natural logarithm of the number of drinks consumed per week in wave *w*. Coefficients from this model can be interpreted as percentage changes in weekly alcohol consumption associated with a 1-unit increase in the number of extreme heat days in a given year. For example, a coefficient of 0.01 indicates that each additional extreme heat day is associated with a 1% increase in the number of drinks consumed per week. *EH_ct_* is the measure for extreme heat events proxied for using the annual counts of days with daily maximum temperatures of 95 °F, 100 °F, and 105 °F in county *c* in year *t*. *X_iw_* indicates a vector of time-variant individual and household characteristics measured in wave *w*. *i_i_* and w*_w_* are individual and wave fixed effects. *i*_*i*_ reflects all the time-invariant individual *i*’s factors such as gender, race or ethnicity, educational attainment, etc. *∈_iw_* is the idiosyncratic error for individual *i* in wave *w*.

The study further explores whether early-life experiences, known to be correlated with alcohol consumption and alcoholism, moderated the association between extreme heat events and alcohol consumption behaviors. The following is the individual fixed effects model after interacting the count of extreme heat days with early-life experience factors:


(2) $$AC_{iw}=\beta_{0}+\beta_{1}EH_{ct}+\beta_{2}EH_{ct}LE_{i}+\beta_{3}X_{iw}+i_{i}+w_{w}+\epsilon_{iw}$$

*LE_i_* denotes the early–life course events of respondent *i*. The early-life experiences included relational quality with a father, police encounters, and parental substance use problems before the age of 18 years. All the life course events were time invariant because the events occurred early in life. In this case, the standalone effects of life course events are absorbed in the individual effects *i_i_*, and the combined effect therefore would be measured as *β_1_*+*β_2_*.

### Ethical Considerations

This study was approved by the institutional review board (IRB) at the University of Utah (IRB Protocol #00194769). All respondents in the HRS provided informed consent prior to participation. The analyses used restricted HRS data containing county-of-residence information, which were accessed under a Restricted Data Use Agreement and analyzed in accordance with applicable data security and confidentiality requirements. No direct participant compensation was provided by the authors because this study relied on the secondary analysis of existing survey data.

## Results

### Extreme Heat Events on Alcohol Consumption Behaviors

Table 2 presents the estimates from individual fixed effects models where the dependent variable was the logarithm of the number of drinks per week. Regardless of the thresholds used to define extreme heat temperatures, the number of extreme heat days was positively correlated with the number of alcoholic beverages consumed per week. A 1-unit increase in the extreme heat day counts defined as the maximum temperatures of 95 °F, 100 °F, and 105 °F related to increases in the number of drinks per week by 0.21%, 0.02%, and 0.04%, respectively. Interpreting the coefficients in more meaningful units, a seasonal increase of 30 extreme heat days (rather than a single day) is associated with a 6.3% increase in weekly alcohol consumption when extreme heat is defined as temperatures ≥95 °F, a 0.6% increase when defined as ≥100 °F, and a 1.2% increase when defined as ≥105 °F.

**Table 2.** The effect of extreme heat on alcohol consumption.^a^

	Dependent variable (log of number of alcoholic drinks/week)		
	Coefficient B (SE)^b^	Coefficient B (SE)^c^	Coefficient B (SE)^d^
Extreme heat days			
≥95	0.0021^e^ (0.0001)	—^f^	—
≥100	—	0.0002^g^ (0.0001)	—
≥105	—	—	0.0004^h^ (0.0001)

a Unweighted individual fixed effects model estimators. 1998 to 2018 waves of the Health and Retirement Study (N=35,261; observations, N=148,696). The covariates include all the factors listed in the *Methods* section.

b *R*2=0.0531.

c *R*2=0.0530.

d *R*2=0.0531.

e *P*<.001.

f Not applicable.

g *P*<.01.

h *P*<.05.

As a sensitivity test, we restricted the sample to respondents who participated in the survey between May and September of each year and reestimated the individual fixed effects models. This procedure addresses the potential concern that the timing of drinking behavior may not align with the period during which extreme heat events are measured. As noted in the *Methods* section, extreme heat is defined using temperature observations from May till September, whereas alcohol consumption is measured throughout the year based on the respondent’s survey date. If the effect of extreme heat is short-lived and primarily influences contemporaneous or near-term drinking behavior—rather than exerting long-lasting effects over the entire year—then restricting the sample to the survey period overlapping with the heat exposure window is appropriate.

The results of this analysis are presented in Table S1 in Multimedia Appendix 1. The estimates are consistent with those from the main specification, although the significance level is reduced to *P*<.05 for models in which extreme heat days are defined as ≥95 °F and ≥100 °F. When extreme heat days are defined as ≥105 °F, the coefficient becomes statistically insignificant. Importantly, the direction and magnitude of the estimated effects remain similar, suggesting that the primary findings are not driven by the inclusion of out-of-season observations. For this reason, we retain the full analytic sample in the main analysis to preserve variation in within-person drinking behavior and maintain statistical power, while presenting the seasonal restriction as a robustness check.

### Early-Life Experiences as a Moderator

From this section onward, the study used a threshold of 95 °F to define the extreme heat days. Table 3 presents the estimates from individual fixed effects models after interacting extreme heat days with early-life experiences (equation 2). All the early-life experiences appeared to moderate the association between extreme heat events and the number of drinks consumed per week. Specifically, early-life relational quality with a father could offset the adverse effect of extreme heat days on alcohol consumption (panel A in Table 3). A 30-day increase in extreme heat days is associated with approximately a 0.6% increase in weekly alcohol consumption among older adults with average levels of paternal relational quality (0.0002 × 30 days = 0.006 or 0.6%). However, for older adults whose paternal relational quality is 1 SD above the mean, this increase is effectively offset. In other words, stronger early-life relationships with fathers buffer the effect of heat exposure on later-life alcohol use. Furthermore, older adults without law enforcement encounters did not change their alcohol consumption in response to changes in extreme heat days (panel B in Table 3). In contrast, among those who experienced adverse police encounters before the age of 18 years, the same seasonal increase in extreme heat is associated with a 2.4% increase in weekly consumption (0.0008×30 days=0.024, or 2.4%). Finally, older adults who did not grow up with parents who had drinking or drug problems show no meaningful change in alcohol consumption in response to additional extreme heat. In contrast, those who did experience parental alcohol or drug problems exhibit a 1.5% increase in weekly alcohol consumption for each 30 additional extreme heat days (0.0005×30 days=0.015, or 1.5%; panel C in Table 3).

**Table 3.** Life course events for alcohol consumption as a moderator.^a^

	Dependent variable (log of number of alcoholic drinks/week), coefficient B (SE)
Panel A: relational quality with a father before the age of 18 years (N=16,018; observations, N=91,701)^b^	
Extreme heat days	0.0002^c^ (0.0001)
Heat × relational quality with a father	−0.0002^c^ (0.0001)
Panel B: police encounter before the age of 18 years (N=13,949; observations, N=47,364)^d^	
Extreme heat days	0.0000 (0.0001)
Heat × police encounter	0.0008^e^ (0.0002)
Panel C: parental drinking or drug problems before the age of 18 years (N=16,081; observations, N=64,291)^f^	
Extreme heat days	−0.0000 (0.0001)
Heat × parental drinking or drug problem	0.0005^e^ (0.0001)

a Unweighted individual fixed effects model estimators. 1998 to 2018 waves of the Health and Retirement Study. The covariates include all the factors listed in the *Methods* section.

b *R*2=0.0570.

c *P*<.01.

d *R*2=0.0731.

e *P*<.001.

f *R*2=0.0565.

## Discussion

### Principal Findings

This study offers novel, population-level insights into the intersection of environmental stressors and behavioral health, specifically examining how extreme heat exposure is associated with alcohol consumption among older adults, with particular attention to the moderating role of early-life experiences. To our knowledge, this is the first analysis within the United States to explore this relationship through a life course framework, highlighting the enduring impact of early ACEs or PCEs on later-life health behaviors.

While some studies have reported potential health benefits of light to moderate alcohol use, such as enhanced cardiovascular function and delayed cognitive decline [36,37], the deleterious consequences of excessive or binge drinking are well established. These include elevated risks of falls, cardiovascular disease, cognitive impairment, and mortality [38], risks that may be exacerbated under extreme heat conditions. Our findings demonstrate a significant association between heat exposure and increased alcohol consumption, aligning with earlier evidence from non-US contexts [39]. However, unlike prior studies, which lacked representative samples and focused on younger populations, our analysis centers on older adults, thereby filling a critical gap in understanding how environmental stress may drive maladaptive coping behaviors in aging populations.

More specifically, our findings indicate that a seasonal increase of 30 extreme heat days is associated with a 1.2% (≥105 °F) to 6.3% (≥95 °F) increase in weekly alcohol consumption. Studies have shown that alcohol consumption during heat exposure can physiologically lower core body temperature by enhancing sweating and vasodilation, which are key mechanisms of thermoregulation [13]. In addition, cold alcoholic beverages may provide immediate psychological relief and a sense of hydration, making them particularly appealing in hot weather [13]. This perceived cooling effect may encourage greater alcohol intake during high temperatures. However, given alcohol’s potential to exacerbate dehydration, impair thermoregulation, and increase vulnerability to heat-related illness [11], it is not a healthy coping strategy during extreme heat. Evidence also shows that higher temperatures are associated with increased hospital visits for alcohol-related disorders [40]. This is especially salient for older adults, as even small behavioral shifts may have clinically meaningful consequences for those already vulnerable to impaired thermoregulation and hydration with aging.

Given the significant health risks associated with excessive alcohol consumption, our findings underscore the need to prioritize populations most vulnerable to both extreme heat and substance use. Targeted interventions for older adults exposed to prolonged heat should consider the compounding effect of environmental stress on health behaviors. Possible strategies include providing subsidies for home retrofitting (eg, air conditioning installation), disaster relief funds, and establishing accessible cooling centers such as community centers, public pools, and shelters. Such measures may not only alleviate heat-induced physiological stress but also reduce the likelihood of maladaptive coping mechanisms such as increased alcohol consumption.

Beyond immediate environmental interventions, this study underscores the enduring influence of early-life experiences on later-life behavioral responses to stress. Specifically, we found evidence for the protective role of PCEs, nurturing, supportive, and emotionally secure environments during early life that contribute to resilience and healthier aging trajectories. In particular, a strong paternal relationship during childhood mitigated the association between extreme heat exposure and alcohol consumption in older adulthood, suggesting that early supportive relationships may foster adaptive coping mechanisms that persist across the life course. Prior research has demonstrated that nurturing paternal relationships buffer against alcohol misuse [21] and enhance stress resilience [20]. Our findings extend this literature by suggesting that the psychological advantages conferred by early positive parenting not only shape interpersonal and emotional health but also influence behavioral responses to environmental stressors later in life. This underscores the value of policies and programs promoting positive parenting practices and mental health education aimed at fostering resilience from childhood through adulthood.

However, the nonsignificant findings for maternal relationships should not be interpreted as an absence of influence (Table S2 in Multimedia Appendix 1). This may reflect limited measurement sensitivity, recall variability, or contextual differences in how maternal warmth and caregiving were reported. Future research should use multidimensional assessments of PCEs that capture diverse caregiving roles and family contexts. Such approaches would better illuminate how early relational environments, across both paternal and maternal domains, can buffer environmental stressors and promote adaptive aging trajectories.

Conversely, the presence of ACEs, including exposure to abuse, neglect, or household dysfunction, was associated with higher alcohol consumption during extreme heat events. This pattern suggests that early-life adversity, particularly exposure to parental substance use, may contribute to enduring susceptibilities in stress regulation and coping behaviors that persist into older adulthood. This finding aligns with previous research highlighting the heightened vulnerability of individuals exposed to ACEs [41]. The influence of parental substance misuse is twofold: it both serves as a behavioral model for maladaptive coping strategies and introduces potential genetic predispositions for substance use disorders [42,43]. These factors collectively increase susceptibility to problematic drinking behaviors in response to stressors such as extreme heat. Addressing these risks necessitates a multifaceted approach—incorporating trauma-informed care, family support programs, and community-based interventions aimed at breaking the intergenerational transmission of substance abuse and fostering healthier coping mechanisms.

Additionally, our findings reveal that negative childhood interactions with law enforcement were associated with increased alcohol consumption in adulthood during heatwaves. Such early encounters often contribute to heightened stress reactivity and may normalize maladaptive coping behaviors within disadvantaged environments [29,44]. This underscores the critical need for trauma-informed policing practices and early intervention programs that mitigate the long-term psychological impacts of ACEs. Integrating trauma-awareness training for law enforcement and expanding mental health services for affected communities could serve as pivotal steps in reducing long-term health disparities.

### Limitations

While this study advances the understanding of the interplay between extreme heat exposure, alcohol consumption, and early-life experiences, certain limitations warrant consideration. The use of HRS data, although robust, restricts our ability to ascertain the precise mechanisms underlying the observed associations. Future research should explore the mediating psychological or physiological pathways in more depth. Additionally, our analysis of PCEs was limited to paternal relationships, as maternal relationships did not yield statistically significant findings in relation to heat exposure and alcohol consumption (Table S2 in Multimedia Appendix 1), highlighting an avenue for further inquiry. An important caveat is that retrospective questions on early-life parental relationships were only administered to respondents who reported having a mother or father present during childhood. Individuals who lacked such parental figures did not receive these items, resulting in nonrandom missingness for that measure. Finally, the absence of detailed environmental variables (eg, home cooling resources and neighborhood infrastructure) represents a limitation, as these factors likely influence the degree of heat-related stress experienced by older adults and shape how they buffer heat-related stress. Future work could incorporate proxy information from ancillary HRS modules such as electricity spending from the Consumption and Activities Mail Survey or community facility measures from the Neighborhood and Network Data to examine potential moderating effects. Although these proxies do not directly capture older adults’ adaptation to extreme heat and linking these subpopulation modules substantially reduces sample size and statistical power, incorporating such environmental factors in future studies will enrich our understanding of the multi-level determinants of health behavior under climate-related stressors.

### Conclusions

This study identifies a clear association between extreme heat events and increased alcohol consumption among older adults, with early-life experiences serving as significant moderators. Positive paternal relationships appear to buffer against the adverse behavioral impacts of heat stress, while adverse experiences such as parental substance use and negative interactions with law enforcement exacerbate vulnerability. These findings emphasize the long-lasting influence of childhood environments on later-life resilience and underscore the need for integrated, trauma-informed policies and interventions. By addressing both environmental stressors and the psychosocial foundations established early in life, policymakers and practitioners can more effectively mitigate the compounding health risks facing older populations in an era of increasing climate volatility.

## Supplementary material

10.2196/76904 Multimedia Appendix 1 Supplementary sensitivity and moderation analyses examining the association between extreme heat exposure and alcohol consumption among older adults.

## Abbreviations

ACE: adverse childhood experience
HRS: Health and Retirement Study
IRB: institutional review board
PCE: positive childhood experience
