# Supplementary material for: Early-Life Experiences as Moderators of the Relationship Between Extreme Heat and Alcohol Consumption among Older Adults: Quantitative Study
Source: JMIR Aging. 2026 Jun 10;9:e76904. doi: 10.2196/76904 (PMC13252698; doi:10.2196/76904)
Supplement: Multimedia Appendix 1 [file aging-v9-e76904-s001.docx]

**Online Supplementary Material**

Table A1. The effect of extreme heat on alcohol consumption (May-September)

|  | DV= log of # of alcoholic drinks/week | | |
| --- | --- | --- | --- |
|  | Coef.  (S.E.) | Coef.  (S.E.) | Coef.  (S.E.) |
| # Extreme heat days ≥ 95 | 0.0001*  (0.0001) |  |  |
| # Extreme heat days ≥ 100 |  | 0.0002*  (0.0001) |  |
| # Extreme heat days ≥ 105 |  |  | 0.0003  (0.0002) |
| R-squared | 0.0543 | 0.0544 | 0.0543 |

*Note*. Individual-fixed effect model estimators. 1998-2018 waves of HRS. N= 28,085. Obs.= 85,037. We restrict the sample to respondents whose survey participation occurred between May and September in each year. The covariates include all the factors listed in the method section. * *p*<0.05.

Table A2. Childhood relationship with the mother as a moderating factor

| ` | DV= log of # of alcoholic drinks/week |
| --- | --- |
|  | Coef.  (S.E.) |
| # Extreme heat days | 0.0001*  (0.0001) |
| Heat × Relational quality with mother | 0.0000  (0.0001) |
| R-squared | 0.0567 |
| N | 15,986 |
| Obs. | 91,576 |

*Note*. Unweighted individual-fixed effect model estimators. 1998-2018 waves of HRS. The covariates include all the factors listed in the method section. * p<0.05
